# The Pathogenesis of Atypical Clinical Presentations of Monkeypox Virus Infections

**DOI:** 10.3390/v18101082

**Published:** 2026-10-01

**Authors:** Lisumi Hewa Ranchigoda Kankanamge, Amal Remtulla, Maedeh Naghibosadat, Emma Wilson, Robert Kozak

**Affiliations:** 1 Sunnybrook Health Sciences Centre, Toronto, ON M4N 3M5, Canada; lhewaran@uwo.ca (L.H.R.K.); amal.remtulla@mail.mcgill.ca (A.R.);; 2 University of Western Ontario, London, ON N6A 3K7, Canada; 3 McGill University, Montreal, QC H2X 3P9, Canada

**Keywords:** monkeypox virus, viral pathogenesis, disseminated disease

## Abstract

Monkeypox virus (MPXV) is an ongoing global public health concern. Historically associated with zoonotic infection, in 2022, a major epidemiological shift occurred when sustained transmission was identified across multiple countries that previously had not had endemic circulation of the virus. While mpox typically presents with characteristic clinical features, infection can also lead to a spectrum of less common manifestations involving multiple organ systems. The extent of viral dissemination and disease severity appear to be modulated by host factors such as immune status, vaccination history, co-infections, and potentially the viral clade. Although complications associated with mpox are infrequent, they can lead to significant morbidity and mortality. Moreover, the underlying pathogenesis and to what extent the viral clade may be playing a role remain incompletely understood. This review summarizes the less common clinical complications of mpox, explores the underlying mechanisms of viral pathogenesis, and discusses what insights can be gained from cell culture and animal models. Additionally, we highlight knowledge gaps that can guide future research priorities.

## 1. Introduction

Monkeypox virus (MPXV) is a zoonotic virus that was first isolated in 1958 in Copenhagen in a colony of cynomolgus macaques, and the first human infection was documented in the Democratic Republic of the Congo in 1970 [1]. Although the definitive animal reservoir remains unknown, several rodent species are considered the leading candidates, whereas non-human primates are likely an intermediate host [2,3]. The first documented outbreak outside its endemic region occurred in the United States in 2003, after imported Gambian pouched rats transmitted MPXV to prairie dogs, which then spread the virus to humans [2,4]. However, in 2022 a major epidemiological shift occurred when sustained transmission was identified across multiple countries that previously had not had endemic circulation of the virus [5]. Unlike earlier outbreaks that were associated with zoonotic or household transmission, the 2022 outbreak was driven by close physical and sexual contact, particularly among men who have sex with men (MSM) [6,7,8]. To date, the global outbreak of Clade IIb monkeypox has resulted in more than 130,000 reported cases worldwide [8].

MPXV is classified into two major clades, I and II, each with distinct geographic and epidemiological characteristics. Clade I, composed of subclades Ia and Ib, is primarily endemic in Central Africa, whereas Clade II, composed of subclades IIa and IIb, has circulated in West African countries. Clade IIb was the primary driver of the large international outbreak that began in 2022, and evidence suggests that it has a reduced severity and case fatality rate (3.6%) when compared with Clade I (10.6%), but potentially a higher transmissibility [8,9]. Experimental studies in animal models have shown differences in virulence, with Clade I causing the most severe disease and highest viral replication, followed by Clade IIa, then IIb [10]. Notably, recent epidemiological investigations in eastern DRC have documented sustained transmission of Clade Ib through sexual and non-sexual contact [11].

## 2. Viral Life Cycle

MPXV is an enveloped, double-stranded DNA virus with a 197 kb genome and a brick-shaped virion approximately 200–250 nm in diameter [12]. Similar to other poxviruses, MPXV can enter cells as either mature virions (MVs) or extracellular enveloped virions (EVs). However, at present, no MPXV-specific entry receptor has been identified. Viral attachment is mediated by multiple viral surface proteins such as A26 (OPG153), A27 (OPG154), D8 (OPG120), and H3 (OPG108) [13]. A26L/A30L (OPG156) binds laminins and supports mature virion entry through endocytic vesicles [8]. Since laminins are extracellular matrix and basement membrane proteins, A26-mediated laminin binding may facilitate MPXV attachment across multiple tissue environments. A26 functions in a complex with A25 (OPG152), A27, and A17 (OPG144), where A17 anchors the complex to the mature virion membrane and A27 mediates the interaction between A26 and A17 [14]. Following attachment, the virus enters the cell through fusion at the plasma membrane or through endocytic pathways, which requires the entry fusion complex (EFC), which promotes membrane fusion to allow the viral core or nucleoprotein complex to enter the host cell cytoplasm [14].

Following entry, the viral particle and core undergo uncoating in the cytoplasm, followed by an eclipse phase during which intact viral particles are not visible, but early viral transcription and virus-mediated modification of the host cell begin [15]. Inside the cell, MPXV replicates in the cytoplasm in viral factories, which are essential for viral transcription, genome replication, protein translation, and assembly of new virions [16]. During the early phase of infection, MPXV reorganizes rough endoplasmic reticulum (rER) membranes into enclosed “mini-nuclei” that are thought to support viral genome replication [15]. In orthopoxvirus models, these structures form within several hours of infection and disappear once virion assembly begins [17]. Similar rER-derived mini-nuclei have been directly observed in MPXV-infected Vero cells [15]. As infection progresses, the mini-nuclei are replaced by larger cytoplasmic viral factories [17]. MPXV virion assembly begins with the formation of crescent-shaped membranes within the viral factory, which acquire electron-dense material from nearby granular regions and eventually close to produce spherical immature virions. Mature MPXV particles are created after the immature virions undergo structural maturation, in which the internal core becomes condensed and organized. Following maturation, MPXV may generate four virion forms: the intracellular mature virion (IMV), intracellular enveloped virion (IEV), cell-associated enveloped virion (CEV), and extracellular enveloped virion (EEV) [18]. Intracellular mature virions may acquire additional membranes from the Golgi complex or related intracellular membrane compartments to form intracellular enveloped virions. These virions are transported toward the plasma membrane and fuse with the host cell membrane, which exposes the particle at the cell surface and forms a cell-associated enveloped virion. Virions that remain attached to the cell surface can promote direct spread to neighboring cells. Virions that detach are released as EEV, although cell culture studies with MPXV studies suggest that these EEVs are less abundant and may appear only during specific stages of the replication cycle [18].

## 3. Clinical Presentation

Viral infection occurs following direct contact, including skin and mucosal membranes, or body fluids, including respiratory droplets. Following an incubation period that ranges from 5–21 days, individuals often present with fever, headache, myalgia, fatigue, and lymphadenopathy. Within 1–2 days of symptom onset, lesions can develop on the skin, oral cavity, or other mucous membranes [19]. The lesions progress through several stages and generally persist for 5–7 days before crust formation occurs. Once the crust is detached, the patient is generally considered no longer infectious [20]. Skin lesions and lymphadenopathy are among the most prominent clinical features of mpox infection, although cases have been reported where no lesions occurred. This may be caused by abnormal proliferation and retention of natural killer (NK) cells, as well as infected macrophages and dendritic cells [21].

Rash characteristics and severity may differ according to viral clade and route of transmission. In Clade I infections, rashes have been reported as being generally monomorphic, although with Clade Ib infections, the nature of the rash remains less clearly defined. In contrast, Clade II infections are more commonly associated with pleomorphic rashes. Genital rashes have been reported across multiple clades but are most frequently observed in Clade IIb infections, although the type of exposure causing infection likely plays a role [22]. Importantly, mpox and varicella–zoster virus (VZV) co-infection has been reported among children and adolescents in Central and West Africa. Several studies have described children presenting with clinical features of both mpox and varicella, with relatively high frequencies of coinfection reported in some study populations. This overlap is clinically important, as the presence of concurrent mpox and varicella may complicate the interpretation of rash morphology and clinical diagnosis in regions where both viruses are endemic. These findings highlight the importance of considering coinfection in children and adolescents presenting with vesicular or pustular rashes in endemic settings [23,24].

Notably, constitutional symptoms of viral illness were reported in 60–85% of patients, respiratory symptoms in approximately 50%, headaches in 30%, oral lesions in 39%, diarrhea in 5%, and ophthalmic manifestations in 6% [25]. Respiratory manifestations may include nasal congestion, sore throat, cough and in some cases shortness of breath [26]. Infection can also result in a range of less frequent clinical manifestations which can affect multiple organ systems. Urogenital involvement is also a common clinical presentation of mpox and can include painful genital lesions such as blisters, vesicles, pustules and ulcers, as well as genital swelling and inguinal lymphadenopathy [27]. Pulmonary involvement may also occur and can be further complicated by coinfections. In immunocompromised individuals, such as those with advanced HIV infection, bacterial or fungal pneumonia may overlap with pulmonary mpox involvement or mpox-associated pulmonary nodules, potentially contributing to increased disease severity [28].

Although the mechanisms underlying disseminated infection remain unclear, it is hypothesized that the virus infects lymphocytes and spreads via the bloodstream, affecting the skin, gastrointestinal tract, liver, kidney, lung, brain, and reproductive organs, while also causing viremia and multi-organ injury and inflammation [21]. Viral dissemination and disease severity are likely influenced by host immune and vaccination status, co-infections, and potentially the viral clade. While the complications associated with mpox infection are relatively infrequent (Figure 1), they have the potential to cause severe morbidity and can even result in death. Herein, we review these complications, provide insight into the underlying pathogenesis, and identify gaps in our collective knowledge where additional research is needed.

## 4. Ophthalmic Disease

Mpox-related ophthalmic infections can affect the ocular surface and anterior segment of the eye and can have a range of different presentations, including conjunctivitis, keratitis, eye lesions, and visual impairment [30,31]. The most common ocular manifestation of MPXV infection is a rash in the periorbital and orbital regions [32]. Mpox ophthalmic disease can be categorized into external and ocular infections. External infection is characterized by the presence of a vesicular rash on the skin of the eyelids and in the periorbital region [31,33,34].

A recent meta-analysis of 25 studies examining mpox ophthalmic disease reported a prevalence of conjunctivitis in 8.9% of cases, keratitis in 3.4%, eye lesions in 3%, and visual impairment in 4.3%. Other ocular manifestations had a pooled prevalence of 12.4% [35]. The virus can reach the eye through either autoinoculation or dissemination as a result of systemic viremia [36,37,38].

Ocular disease is not unique to MPXV and have been observed with other poxviruses including variola virus (VARV), cowpox virus, and vaccinia virus [36]. All can affect nearly every part of the eye, from the eyelids to the conjunctiva and cornea, and sometimes the posterior segment [36]. Historically, VARV produced distinctive rashes around the eye, along with swelling and discharge, and pustules on the conjunctiva that cause pain, photophobia, and excessive tearing [36]. Cowpox virus has been associated with similar presentations, including eyelid swelling, ulceration, conjunctivitis, and superficial keratitis.

Conjunctivitis due to MPXV infection may manifest as conjunctival ulcers, disseminated blistering or papular conjunctival lesions, conjunctival follicular reactions, and pseudomembranous or subconjunctival nodules [32]. Among these, the follicular reaction was the most frequently reported (54.5%) in a case study of 11 people [39]. Mpox patients who had conjunctivitis also reported more frequent systemic symptoms (such as nausea, chills and sweats, and lymphadenopathy) than those without conjunctivitis [32]. Children tend to be more susceptible to conjunctivitis associated with MPXV infection [32].

Keratitis, particularly ulcerative keratitis, can lead to corneal scarring and significant visual impairment [40]. Keratitis from MPXV infection is frequently protracted, and corneal involvement substantially increases the risk of lasting visual impairment [40]. Corneal inflammation may persist for months despite treatment. In one immunocompetent patient, peripheral ulcerative keratitis was associated with corneal inflammation lasting more than six months across repeated antiviral courses [40], as well as persistence of up to eight months [41]. Outcomes of keratitis vary from full recovery to no light perception [41]. MPXV can produce disciform keratitis and scarring closely resembling other ocular viral infections, specifically ocular vaccinia and herpes simplex virus type 1 (HSV-1) disciform keratitis, which complicates clinical diagnosis.

Eyelid and periocular disease are also frequently reported in ocular manifestations, ranging from eyelid edema and pustular or nodular lid lesions to blepharitis, preseptal (periorbital) cellulitis, and, in severe cases, necrosis of the palpebral skin [42]. Additionally, blepharoconjunctivitis has been reported as a presenting feature of infection, and a severe necrotizing blepharoconjunctivitis has been described in an HIV-positive patient, underscoring that adnexal involvement can be sight-threatening rather than merely superficial [43]. Scleral involvement, including scleral edema and episcleritis, has also been reported [42]. Additionally, uveitis has been described in both systemic and isolated ocular mpox, and one severe report documented concurrent fulminant scleritis, keratitis, and uveitis in a patient whose aqueous humor tested positive for MPXV DNA, confirming intraocular viral presence [44]. Furthermore, posterior segment involvement such as retinitis or vitritis has not been a feature of recent outbreaks, in contrast to the intraocular complications historically described with VARV [40]. It is unclear if infectious MPXV is present in tear drops, as has been described with VARV, but this should be investigated to evaluate the potential for transmission [45].

Epidemiological data suggests that the frequency and severity of ocular manifestations differ between MPXV clades. The reported prevalence of ocular involvement ranges from roughly 0.3–11% in Clade II infections to 4.1–23.1% in Clade I infections, and Clade I is also associated with higher rates of conjunctivitis and visual impairment [46,47]. An umbrella review of 36 primary studies that included 28,139 patients reported a comparable overall conjunctivitis prevalence of 8.9% but, critically, stratified outcomes by clade [47]. Ocular involvement was consistently more frequent and more severe in Clade I. Conjunctivitis prevalence reached 21.9% in Clade I compared with 2.7% in Clade IIb, and unilateral (0.9%) and bilateral (0.4%) blindness were reported only in Clade I infections. Across the included reviews, people living with advanced HIV experienced disproportionately higher rates of severe and persistent ocular complications than HIV-negative individuals [47]. This difference has been noted in previous outbreaks as well, during the 2014 Clade I outbreak, where conjunctivitis was reported in 23.1% of cases [31].

The pathogenesis underlying the ocular complications of MPXV infection remains unclear. However, two possible mechanisms have been suggested based on observations. This includes self-transmission from adjacent eyelid margin lesions and the development of both eyelid margin and conjunctival lesions as part of regional infection spread [32]. Historical studies of vaccinia and VARV suggest that autoinoculation is the most likely cause of ocular complications [45]. However, this is based heavily on vaccination studies with vaccinia, and it is unclear if the different routes of transmission seen in the current mpox epidemic may play a role. Moreover, the observed difference in ocular disease between Clade I and Clade II make it tempting to speculate that clade-specific viral factors may play a role. While non-human primate studies observed necrotizing conjunctivitis following aerosol exposure to Clade I virus, animal challenge studies that used a Clade IIb virus and mimicked natural routes of infection also reported conjunctivitis [32,48]. As these studies did allow for follow-up of the animals after recovery from the acute phase of infection, it is not known how viral clade, route of transmission, or infectious dose may affect ocular complications. Further cell culture and organoid studies are needed to better elucidate the mechanisms of viral pathogenesis of both clades of MPXV.

## 5. Neurological Complications

Although headaches and myalgia are common symptoms of mpox infection, a wider range of other neurological complications have also been reported [49]. A systematic review of 22 studies of mpox patients reported headache (48.8%) and myalgia (27.5%) as the most commonly manifestations but noted that encephalitis (0.8%), seizures (0.3%), encephalomyelitis (0.2%), coma (0.1%), and transverse myelitis (0.1%) also occurred [49]. A case series of 18 mpox patients who developed neuroinflammatory disorders reported that while disseminated encephalomyelitis (50%) and encephalitis/meningoencephalitis were the most common complications (50% and 38.9% respectively), isolated transverse myelitis (5.6%), and transverse myelitis with encephalitis (5.6%) were also seen. Notably, the mean age in the cohort was 27.8 years (range of 28 days to 43 years) highlighting that neurological sequelae can affect all age groups. Comorbidity status was documented in 13 cases (72.2%). Co-infections were identified in a third of the cases (n = 4 syphilis, n = 2 HIV), making it unclear to what degree these may be contributing to this neurological complication [50]. Interestingly, viral encephalitis has been reported for MPXV and VARV, but not for vaccinia virus, making it tempting to speculate that viral factor unique to these viruses may account for this pathology [51].

The clinical severity of CNS involvement associated with MPXV was demonstrated in two previously healthy young men who developed encephalomyelitis during the 2022 outbreak [52]. Neurological symptoms appeared shortly after the onset of systemic disease, developing five days after symptom onset in one patient and nine days after symptom onset in the other. This temporal relationship suggests that neurological injury can develop during the acute stage of MPXV infection rather than occurring only as a late complication. Spinal imaging showed longitudinally extensive lesions involving the central thoracic spinal cord and the grey matter of the conus medullaris, demonstrating that MPXV-associated encephalomyelitis may affect several anatomically distinct regions of the brain and spinal cord simultaneously. The second patient initially developed bowel and bladder incontinence and progressive muscle weakness of both lower extremities. His neurological condition rapidly progressed to altered mental status, requiring intubation and intensive care admission. Brain imaging of the second patient demonstrated lesions involving the pons, cerebellum, and medulla, while spinal MRI revealed multifocal, partially enhancing lesions in the central cervical and upper thoracic spinal cord. Interestingly, MPXV DNA was not detected in the CSF of either patient, although MPXV infection was confirmed in skin lesions. The neurological injury may have also resulted from the immune response rather than sustained viral replication in the CSF [52].

Rare peripheral nervous system complications have also been described following monkeypox infection. Two separate case reports documented Parsonage–Turner syndrome (PTS), also known as neuralgic amyotrophy, associated with mpox. PTS is a rare inflammatory peripheral neuropathy affecting the brachial plexus. Although its exact pathophysiology is not fully understood, viral infections have been reported as triggers in approximately 20–52% of cases, while vaccination has been reported as a trigger in approximately 15% of cases [53]. The proposed mechanism of infection involves a post-infectious immune-mediated inflammatory response that damages peripheral nerves, although direct viral infection of nerve tissue has also been proposed as a possible mechanism in mpox [53]. One case report described Parsonage–Turner syndrome (PTS) in a man who developed severe shoulder pain, weakness, and muscle wasting approximately two weeks after MPXV infection. Five months later, the patient reported that weakness continued to interfere with daily life, and examination showed visible atrophy of the muscles in the arm and shoulder [53].

In another reported case, a previously healthy 40-year-old male was infected with MPXV seven days after he received the first dose of the JYNNEOS vaccine [54], and he subsequently developed severe bilateral shoulder pain and spasms and weakness in external rotation of the left shoulder. Six weeks later, examination showed visible atrophy of the left supraspinatus and infraspinatus muscles. Because infection and vaccination occurred close together, it could not be determined whether MPXV, vaccination, or a combined immune response triggered the neuropathy. The close timing of PTS onset was considered more consistent with an infection-associated trigger because most infection-related cases develop within seven days of the preceding illness, whereas vaccination-associated PTS more commonly develops approximately 17–28 days after vaccination [54].

Multiple mechanisms may explain how MPXV is able to infect the CNS. Following intranasal MPXV inoculation in ground squirrels, high viral loads and active replication were detected in the nasal mucosa, suggesting that tissues near the olfactory pathway may represent a potential entry point for viral spread toward the central nervous system [55]. Furthermore, imaging studies in mice following intranasal inoculation of MPXV showed virus replication in the intranasal and brain areas [56]. Furthermore, poxviruses are known to exploit actin tails and tunneling nanotubes to facilitate spread [57]. In vaccinia virus-infected cells, infection induced the formation of membrane nanotubes which were developed through previously connected cells that moved apart while remaining joined, a protrusion from one infected cell that extended toward another cell, or protrusions from two separate cells that met and formed a connection. Because membrane nanotubes can act as physical connections between cells and facilitate the transport of intracellular cargo and pathogens, their induction by orthopoxvirus infection may provide an additional pathway for direct cell-to-cell viral dissemination [57]. Thus, the MPXV may directly infect the olfactory epithelium and then travel along olfactory nerve pathways through axonal transport, allowing the virus to reach the brain parenchyma. However, the 2022 epidemic has highlighted other routes of infection, suggesting that viral dissemination to the CNS likely relies on the hematogenous route whereby infected monocytes and macrophages act as viral reservoirs to allow the virus to cross the blood–brain barrier [58]. Human studies provide evidence consistent with systemic dissemination through the bloodstream during acute mpox. In an observational study of 162 patients, MPXV DNA was detected in plasma in 65% of patients at diagnosis [59]. Patients with detectable MPXV DNA in the bloodstream more frequently exhibited disseminated non-oral/non-genital skin lesions (83% vs. 67%) and systemic manifestations, including fever (58% vs. 42%) and lymphadenopathy (57% vs. 40%), compared with patients without detectable bloodstream viral DNA [59]. A longitudinal study detected MPXV DNA in 62.3% of plasma, 49.2% of serum, and 37.7% of whole-blood samples collected during the first three weeks following symptom onset, with plasma demonstrating the highest viral DNA detection rate and viral load among the three blood sample types [60]. Hematogenous dissemination may therefore represent one potential route by which MPXV gains access to distant tissues, including the CNS. Cell culture and organoid experiments provide insight into mechanisms of MPXV pathogenesis once it has invaded the CNS. In vitro infections of primary human astrocytes with a Clade IIb MPXV isolate observed reductions in pro-caspase-3 and GFAP following infection, suggesting that activation of caspase-3 may contribute to GFAP degradation and the dysmorphic appearance of infected astrocytes. Because astrocytes are abundant CNS cells that support neuronal function and contribute to maintenance of the local neural environment, their structural disruption and death may contribute to MPXV-associated neurological disease [61]. The same study found that the MPXV and vaccinia virus infection increased expression of TNF-α, IL12, IRF3, IL1B, CASP1, and GSDMB, which are associated with inflammation, antiviral defense, and inflammasome activity. However, only MPXV caused marked plasma–membrane rupture and lytic astrocyte death. MPXV infection also activated the inflammasome-associated protease caspase-1 and induced cleavage of gasdermin B, a primate-specific pore-forming protein that is abundantly expressed in human astrocytes. Gasdermin B cleavage releases a pore-forming N-terminal fragment that disrupts the plasma membrane, releases inflammatory cytokines, and triggers pyroptosis. Heat-inactivated MPXV failed to produce detectable I3 protein or gasdermin B cleavage, indicating that active viral infectivity and replication were required to initiate this pyroptotic pathway [61].

A study analyzing the susceptibility of neural tissue to MPXV using human neural organoids (hNOs) infected with Clade IIb demonstrated that the virus can infect multiple neural cell types, including neural progenitor cells, neurons, and astrocytes. The virus can also replicate efficiently within neural tissue and spread through cell-to-cell transmission along neurites [62]. The same study detected the MPXV A27L antigen within neural cell bodies and neurites, indicating that viral material was present inside the cells. A27L is a conserved, multifunctional envelope-associated protein involved in poxvirus attachment and membrane fusion. A27L also contributes to viral movement through interactions with microtubule-associated transport systems. In the CNS, neurons are highly connected, so this characteristic allows MPXV to move locally through connected neural networks. In neurons, MPXV was observed inside thin cellular extensions, including neurites, and infected hNOs revealed clustered infection foci indicating cell-to-cell spread [62]. Condensed cytoskeletal structures were frequently observed near viral factories within infected organoid cells. Since orthopoxviruses can manipulate both actin and microtubule-based transport pathways, it has been proposed that MPXV may use the neuronal cytoskeleton and fast axonal transport machinery to move intracellular virions through axons and dendrites. Such a mechanism could allow the virus to cross directly into connected cells and reduce its exposure to extracellular antibodies and other antiviral factors. As MPXV accumulates in the neurites, its structure becomes disturbed and causes neuritic beading, meaning swollen bead-like structures formed along infected neurites. This characteristic causes neuronal dysfunction and could be an underlying mechanism of the clinical symptoms seen in patients exhibiting neurological manifestations. In live-cell imaging, neuritic beading appeared before or around the same time as neuronal degeneration [62]. The study also found occasional cleaved caspase-3, which suggests apoptosis may be involved, but the cell death pathway is not fully understood [62]. Furthermore, MPXV infection altered the transcriptome of hNOs. The most relevant changes were in genes related to synaptic organization, trans-synaptic signaling, neurodegeneration-associated transcripts, and cellular homeostasis [62]. Genes related to basic cellular functions were also downregulated, while there was an increase in inflammatory and antiviral mRNAs, including IL-6, IL-8/CXCL8, IP-10/CXCL10, and IFN-β. Despite the increase in mRNA, the protein response was low or undetectable for many of these cytokines, suggesting that monkeypox can suppress the host antiviral response to allow viral replication and spread before the tissue mounts a strong defense [62]. The virus also produces proteins that interfere with host immune defenses by modulating apoptosis, reducing antigen presentation through MHC-I, and mimicking host immune-regulatory molecules that bind cytokines [62].

## 6. Psychiatric and Post-Viral Complications

Despite in vitro and in vivo findings that indicate MPXV causes neuropathology, data on psychiatric sequelae is often not captured in reviews of neurological disease. Psychiatric sequelae have also been noted in a subset of mpox patients. A systematic review and meta-analysis by Badenoch et al. of 19 studies with 1512 participants, of whom 1031 had confirmed MPXV infection, noted that anxiety, depression, low mood, emotional lability, distress, and suicide were reported [63]. However, Badenoch et al. emphasized that psychiatric symptoms were not assessed using standardized psychiatric scales in the included studies, and long-term psychiatric outcomes remain poorly characterized [63]. Ogonia et al. reported anxiety and depression in over one-quarter of hospitalized patients with monkeypox in Nigeria [64]. The psychiatric burden of mpox is especially important because the disease can be highly visible and socially stigmatizing. Stigma may also worsen anxiety, depression, and social isolation. The 2022 Clade IIb outbreak was mainly driven by close physical and sexual contact, particularly among MSM, placing greater harm on the LGBTQ+ community [6,7,63]. Psychiatric and mental health issues have been reported in other post-encephalitis cases, and further research is needed to determine the degree to which pathogenesis of the virus may contribute to this condition [65]. Post-viral syndromes have been reported for numerous viruses including influenza virus, Epstein–Barr virus, Ebola virus, West Nile virus, and SARS-CoV-2, and it is possible that a similar syndrome may occur in individuals recovering from mpox; in fact, chronic pain and urinary dysfunction have been documented. As another salient example, Parsonage–Turner syndrome (PTS) was reported in a vaccinated individual following MPXV infection; as PTS can occur weeks to months after a viral infection, it may be more prevalent but under-reported, or it may not be easily linked to a previous mpox diagnosis [54]. In the case of post-viral syndrome from the Life After Mpox cohort, 154 adults with laboratory-confirmed Clade IIb infection were assessed [66]. Of that cohort, 58% retained at least one persistent physical complication (most commonly mild appearance-related changes such as scarring or discoloration), while 13% reported functional complications including anorectal and urinary symptoms [66]. Persistent psychosocial effects were similarly frequent, with mpox-related stigma manifesting as emotional distress (30%), social avoidance (22%), and employment loss or denial (18% and 8%, respectively) [66]. These findings blur the boundary between post-viral and psychiatric sequelae and suggest that the long-term burden of mpox is as much psychosocial as physical. Nevertheless, available longitudinal data remain confined to a small number of predominantly Clade IIb, MSM cohorts, and standardized prospective assessment across clades and populations will be required to establish whether a discrete post-viral syndrome exists.

## 7. Cardiovascular Complications

Myocarditis, pericarditis, and myopericarditis have been reported as rare cardiovascular complications of MPXV infection [67]. A review of 528 cases across 16 countries reported only 2 cases of mpox-associated myocarditis, suggesting a low prevalence rate [67]. Amongst the case series of mpox-associated cardiovascular complications, myocarditis is the most frequently reported condition, although pericarditis and myopericarditis have also been reported [68]. The case series noted that all patients were male and between 21 and 52 years of age, and several patients were either living with HIV (4 patients) or using HIV pre-exposure prophylaxis (5 patients). Cardiac involvement tended to occur early, with a median onset of 5 days after initial symptoms [68]. The most common symptom was chest pain, which was reported in 17 of 20 patients from the study by Halani et al. Information on symptom recovery was reported for 9 patients. Six patients improved within 1 to 3 days after hospital admission, two recovered by follow-up assessments conducted approximately 1 month and 1.5 months later, and one recovered before discharge on day 19. Follow-up cardiac MRI was performed in two cases at 4 and 8 weeks, respectively, and both showed normal results. No deaths have been reported from mpox-associated myocarditis or pericarditis [68]. Further clinical evidence was provided by a 2022 case report describing MPXV-associated myocarditis in two previously healthy men in the United States who had not received an orthopoxvirus vaccine [69]. The first patient, a 32-year-old man, developed chest pain and shortness of breath seven days after the onset of headache, fatigue, and cervical lymphadenopathy and approximately two days after the appearance of the rash. Laboratory testing indicated myocardial injury, but his electrocardiography showed normal sinus rhythm. He received tecovirimat for mpox but did not require myocarditis-specific treatment. The second patient was a 37-year-old man who developed dyspnea and reduced exercise tolerance approximately six days after the onset of mpox symptoms and four days after the rash appeared. Electrocardiography showed T-wave inversions in the inferior and anterolateral leads, although these abnormalities subsequently improved. Echocardiography showed normal biventricular size, systolic function, regional wall motion, and age-appropriate diastolic function. His dyspnea resolved by the fourth hospital day, and cardiac enzyme levels normalized without treatment for mpox or myocarditis. Interestingly, these cases suggest that mpox-associated myocarditis can produce symptomatic and biochemical evidence of myocardial injury even when ventricular function is normal and structural abnormalities are not detectable by echocardiography [69]. The pathogenesis underlying the cardiovascular complications of monkeypox remains unclear, but there are several proposed mechanisms. The virus may directly infect vascular endothelial cells, which are important for maintaining normal blood vessel functioning [70,71]. Viral injury to these cells can cause endothelial dysfunction and cardiovascular complications [72]. This is supported by studies in cynomolgus macaques, where MPXV DNA was detected following infection with aerosolized MPXV [73]. However, it should be noted that these animals were infected with a Clade I virus, and NHP challenge studies that used a Clade IIb virus did not report any cardiac complications [74]. To date, no human studies on infections with MPXV Clade IIb have reported viral isolation or viral antigens from biopsy samples, although the difficulty in sample collection must be taken into consideration. Additionally, there is a paucity of reports on myocarditis arising following Clade I infections in humans, despite the increased virulence in NHPs, although limitations in epidemiological studies in regions where Clade I is circulating may be a factor.

MPXV can cause cellular death by directly invading and replicating within the myocardium. In vitro studies in human embryonic stem cells showed the MPXV H3L downregulates cardiac genes involved in cardiac development and morphogenesis [75]. Additionally, H3L upregulated IL-1A, inducing apoptosis and cell death, thus indicating a potential mechanism of viral pathogenesis. Tissue damage could also be a result of the host’s immune response to MPXV, potentially following clearance of the virus. This would likely be similar to what has been observed in individuals who have received the smallpox vaccine, where histopathology demonstrated an influx of inflammatory cells, but no vaccinia virus DNA was detected [76]. During MPXV infection, inflammatory cytokines such as IL-6 and TNF-α can activate endothelial cells and cytotoxic T-cells that promote inflammation of cardiovascular tissues [77,78]. Similarly, the pathogenesis of mpox-associated pericarditis involves direct viral invasion and immune-mediated mechanisms. MPXV can directly invade the pericardium and cause local inflammation. The immune response triggered by the viral infection can cause an inflammatory reaction in the pericardium, causing the release of pro-inflammatory cytokines and the infiltration of immune cells into the pericardium [79]. Further studies are needed to determine the relative contributions of direct infection and the host response to pathogenesis. Clinically, the extent to which viremia, viral load, and the route of infection influence MPXV dissemination to cardiac tissue remains to be elucidated. Finally, there is currently no definitive data comparing the specific prevalence of myocarditis between Clade I and Clade II mpox virus.

## 8. Gastrointestinal Complications

Gastrointestinal manifestations in mpox patients have been documented in multiple studies spanning decades and across different countries regardless of viral clade or route of transmission (e.g., human-to-human or zoonotic). Common manifestations include vomiting, diarrhea, rectal pain, nausea, tenesmus, rectal bleeding, and abdominal pain [80]. Painful defecation, rectal perforation (RPE), and proctitis have also been noted, with proctitis reported as a predominant manifestation in a significant proportion of patients [80,81]. A review of 31 studies further quantified these findings, reporting that anorexia was the most prevalent (47%), followed by vomiting (12%), nausea (10%), abdominal pain (9%), and diarrhea (5%), while proctitis (11%), rectal/anal pain (25%), and rectal bleeding (12%) were also reported [80,81,82].

Variation in GI symptoms has been observed across populations, with abdominal pain appearing to be a more common in African studies than non-African ones [25]. Non-African and adult-only cohorts show lower GI symptom burden, suggesting that GI involvement may be more associated with pediatric populations and that the viral clade may play a role [81]. When comparing pathophysiology between children and adults, children may show different (often higher) rates or severity of mpox gastrointestinal symptoms compared with adults, potentially due to differences in innate and adaptive immune responses [81]. Outbreaks in African countries have involved MPXV Clade I, which may have virulence or tissue tropism differences that could be contributing to the reported GI symptom prevalence, particularly abdominal pain [81]. By contrast, in the 2022 outbreak which involved Clade IIb, rectal pain was reported in 16.82% of cases, while dysphagia ranged from 2% to 70.08%, with pharyngitis/dysphagia combined at 23% [25]. Additionally, it is unclear whether anorexia is a direct effect of the mpox virus or secondary to other manifestations such as nausea and vomiting [80].

The pathogenesis underlying these GI manifestations includes the possibility that the virus could infect rectal mucosal epithelial cells, specifically enterocytes and goblet cells, through cell-surface receptors such as chondroitin sulfate or heparan sulfate, which bind to viral surface proteins such as MPXV A29 [80,83,84]. Non-human primate challenge studies have shown multiple cell types in the stomach, cecum, ileum, colon, and rectum. Interestingly, this was following an intranasal challenge, suggesting hematogenous dissemination, likely by infected macrophages.

Infection studies using colon organoids show that virulence differs between Clade I and Clade II viruses; it was observed that the MPXV Zr-599 stain (Clade I) grew to higher titers than to a 2022 MPXV Clade IIb virus [85]. Furthermore, RNA-sequencing showed that MPXV Zr-599 infection dysregulated genes involved in zinc homeostasis, which could help explain the greater likelihood of GI symptoms reported by multiple African studies. Further studies are needed to determine if one or more genes unique to Clade I viruses directly facilitate this.

Interestingly, proctitis was identified as a novel sequela in the 2022 outbreak, although this may be a result of direct inoculation of the virus into the rectal mucosa during receptive anorectal intercourse, as has been shown for other STIs [86,87]. The mechanism of viral pathogenesis likely involves direct viral invasion of rectal tissue by MPXV DNA, and viruses have been isolated from rectal samples as well as systemic cytokine-mediated inflammation [88]. Patients with proctitis often also have early systemic symptoms before developing skin lesions [82]. It has been observed that HIV co-infection has also been associated with a higher likelihood of complications such as proctitis, and further work is needed to determine if co-infection with other STIs also increases the risk [87].

## 9. Hepatitis

Interestingly, hepatic involvement following MPXV infection appears to be relatively rare in the 2022 mpox outbreak as a study of 528 patients from 16 countries did not report any instances of hepatitis [67]. However, liver biopsies from six fatal cases in immunocompromised patients revealed hepatic necrosis in 50% of the patients [89]. Viral clade may play a role in hepatic involvement, as a review of recent cases from the Democratic Republic of Congo, where Clade I is circulating, noted hepatomegaly in 7.9% of patients, although it did not report liver enzyme levels [90].

Additionally, a review of 34 cases from the 2003 outbreak in the USA noted hepatitis in 3% of the cohort, although coinfection with Hepatitis C virus was also noted [91].

Since 2022, there has been a paucity of case reports of viral hepatitis, and these have been amongst individuals co-infected with HCV or HBV [92]. In the case of HCV, it remains unclear if co-infection results in increased disease severity, as clinical reports suggest that HCV/mpox has less severe pathological changes than expected in mpox or HCV alone and may be associated with a less severe inflammatory skin response [93]. Route of infection may play a role in MPXV infection of the liver, as 3 out of 15 cynomolgus macaques showed histopathological evidence of hepatitis following an aerosol infection with a Clade Ia isolate. Analysis of liver samples indicated hepatocytes and Kupffer cells were infected, and viruses were able to be isolated [48]. This differs from what has been observed in fatal human cases, where MPXV antigen was only detected in hepatocytes but not Kupffer cells. By contrast, infection of cynomolgus macaques with Clade IIb by different routes (intraocular, intrarectal, and intradermal) did not result in any evidence of hepatitis [74,89]. Future studies should examine the role of co-infection with other causes of viral hepatitis in disease pathology as well as how route of infection, clinical and viral factors may influence the likelihood of liver sequelae.

## 10. Co-Infections with Mpox

Co-infections are a significant mpox complication, with reported cases involving HIV, syphilis, HSV-1, HSV-2, HBV and VZV. These co-infections may complicate diagnosis and clinical management and potentially influence disease severity. However, their contributions to mpox virulence remain unclear. HIV and syphilis have been reported in patients with neurological complications of mpox, with coinfections identified in approximately one-third of reported cases, making their contribution to neurological disease difficult to determine [50]. HIV co-infection has also been associated with an increased likelihood of proctitis [87]. The impact of co-infection with STIs other than HIV on MPXV-related complications must also be investigated. A review of cases from 16 countries highlighted that 29% of individuals enrolled in the study had a non-HIV STI, with syphilis being the most frequent co-infection. Whether or not HIV or other STIs predispose individuals to having MPXV disseminate to a particular organ remains a topic for further study [67]. Viral hepatitis has similarly been reported in individuals coinfected with HBV or HCV [92]. However, whether HCV co-infection increases disease severity remains unclear, with some reports suggesting less severe pathological and inflammatory changes in HCV/mpox co-infections. Co-infection with VZV has been reported particularly among children and adolescents in Central and West Africa, with some studies reporting substantial rates of coinfection. This overlap may complicate the clinical interpretation of vesicular and pustular rashes [92]. Additionally, HSV-2 coinfection has been reported in an HIV-positive patient presenting with perianal lesions, highlighting the potential for overlapping mucocutaneous manifestations and the importance of considering additional sexually transmitted infections in patients with suspected mpox [94]. In immunocompromised individuals, particularly those with advanced HIV infection, concurrent bacterial or fungal infections may further complicate mpox, including overlapping pneumonia in patients with pulmonary involvement [28]. Overall, co-infections may contribute to inflammation, organ injury, or disease severity; however, further research is required to determine the extent to which specific pathogens directly influence mpox pathogenesis and clinical outcomes.

## 11. Conclusions and Future Directions

Mpox will continue to be an ongoing threat to global public health; surges in cases and the introduction of the virus into new populations are certainly possible. As such, there is an urgent need to better understand MPXV pathogenesis and its less common clinical manifestations. The 2022 epidemic has identified previously under-appreciated mechanisms of transmission, and it remains unclear what role, if any, the route of transmission plays in pathogenesis and related complications. More data is needed on how vaccination or previous infection reduces the likelihood of complications following monkeypox infection. A recent global case series noted that disease course was shorter in re-infected patients, and none were hospitalized, nor were complications reported [95]. However, this was a relatively small cohort (n = 37), making it difficult to draw definitive conclusions. Further work is also needed to determine if sex contributes to a greater likelihood of complications, and if so which ones. This paucity of data may be due to fewer clinical reports coming from endemic areas, as a 2024 systematic review determined that these countries reported higher proportions of women who had monkeypox compared to studies from non-endemic countries (43.13% vs. 6.15%) [63].

Non-human primate models have been shown to model human disease, and these should be further utilized to address the above-mentioned knowledge gaps. Furthermore, employing cutting-edge technologies and assays (e.g., RNA-seq, proteomics, etc.) on banked patient samples may also provide insight. A deeper understanding can help improve patient treatment and guide the development of novel therapeutics.

These research priorities align with the World Health Organization’s framework for strengthening preparedness and response to public health emergencies. WHO emphasizes five core response strategies: coordination, surveillance, community protection, care, and countermeasures [96]. Continued investigation of MPXV pathogenesis and clinical complications can strengthen these pillars by improving disease surveillance, informing clinical care, and supporting the development of vaccines and other treatments. Integrating advances in MPXV research with these broader public health strategies will be important for limiting disease burden and improving preparedness for future outbreaks.

## Figures and Tables

**Figure 1:**
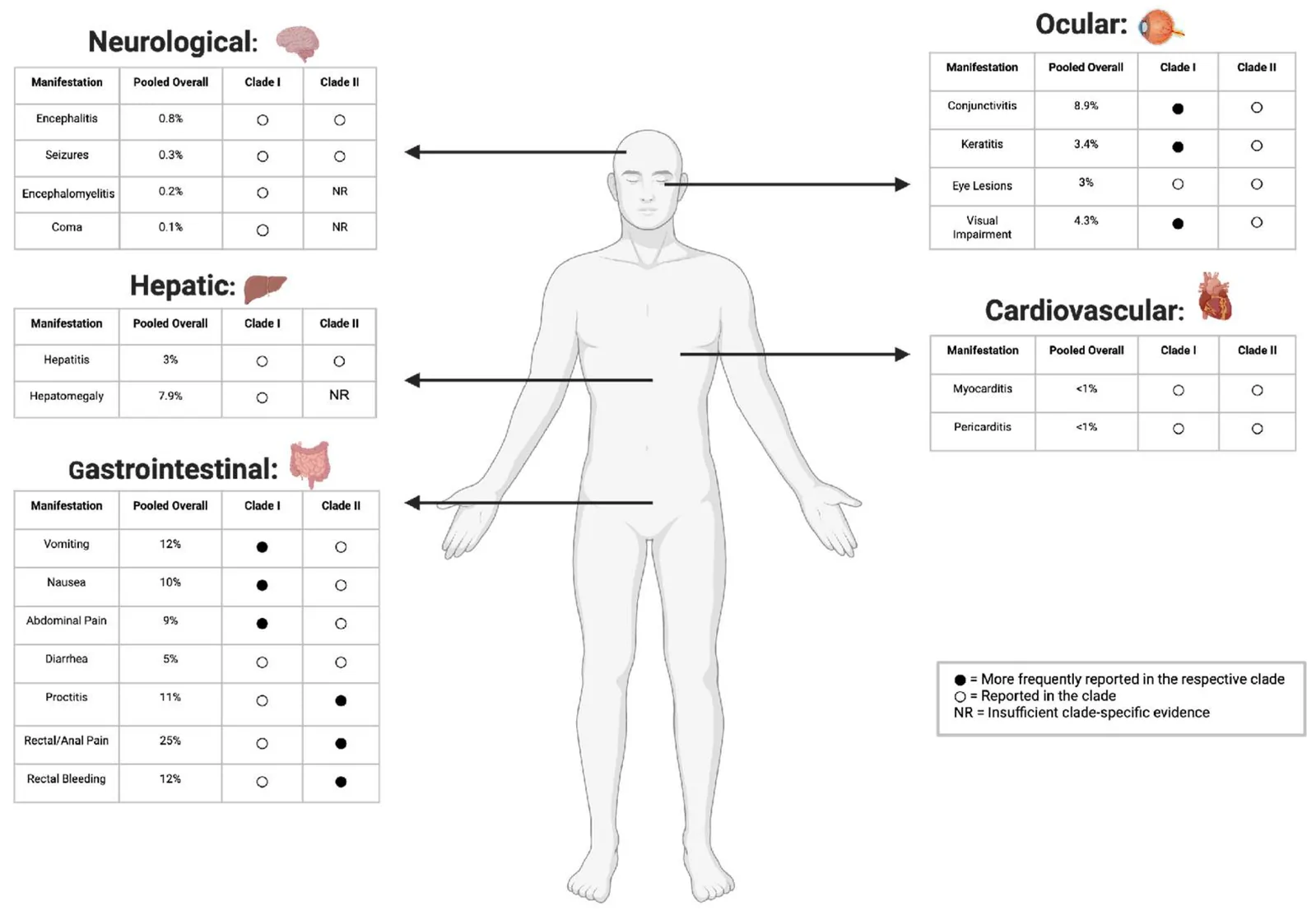
Multi-system clinical manifestations reported in patients with mpox. Clinical manifestations in Clade I and Clade II mpox infections shown across neurological, gastrointestinal, hepatic, ocular, and cardiovascular systems. Pooled overall prevalence estimates for individual manifestations are displayed alongside each manifestation and have been derived from heterogenous study designs. Black circles (●) indicate manifestations more frequently reported or more strongly associated with the respective clade, white circles (○) indicate manifestations reported in the respective clade. NR indicates insufficient clade-specific evidence [29].

## Data Availability

No new data were created or analyzed in this study. Data sharing is not applicable to this article.

## Abbreviations

The following abbreviations are used in this manuscript:

MPXV	Monkeypox virus
OPG	Orthopox virus gene
CNS	Central nervous system
VARV	Variola virus
STI	Sexually transmitted infection
PTS	Parsonage–Turner syndrome
HCV	Hepatitis C virus
HBV	Hepatitis B virus
HIV	Human immunodeficiency virus
hNOs	Human neural organoids
NHP	Non-human primate
